# Renal Dysfunction Is an Independent Risk Factor for Poor Outcome in Acute Ischemic Stroke Patients Treated with Intravenous Thrombolysis: A New Cutoff Value

**DOI:** 10.1155/2017/2371956

**Published:** 2017-01-03

**Authors:** Elyar Sadeghi-Hokmabadi, Demet Funda Baş, Mehdi Farhoudi, Aliakbar Taheraghdam, Daryoush Savadi Oskouei, Mohammad Yazdchi, Maziyar Hashemilar, Nevzat Uzuner, Reshad Mirnour, Ertugrul Colak, Atilla Özcan Özdemir

**Affiliations:** ^1^Department of Neurology, Neurocritical Care, Cerebrovascular Disease, Eskisehir Osmangazi University, Eskisehir, Turkey; ^2^Department of Neurology, Neuroscience Research Center (NSRC), Imam-Reza Hospital, Tabriz University of Medical Sciences, Tabriz, Iran; ^3^Department of Neurology, Cerebrovascular Disease, Eskisehir Osmangazi University, Eskisehir, Turkey; ^4^Department of Biostatistics, Eskisehir Osmangazi University, Eskisehir, Turkey

## Abstract

*Objective*. This study was set to assess the effect of renal dysfunction on outcome of stroke patients treated with intravenous thrombolysis (IVT).* Methods*. This multicenter research involved 403 patients from January 2009 to March 2015. Patients were divided into two groups: (1) control group with GFR ≥ 45 mL/min/1.73 m^2^ and (2) low GFR group with GFR < 45 mL/min/1.73 m^2^. Outcome measurements were poor outcome (mRS 3–6) and mortality at 3 months and symptomatic intracerebral hemorrhage (SICH) within the first 24–36 hours. Univariate and multivariate regression analyses were performed, and odds ratios (ORs) were determined at 95% confidence intervals (CIs).* Results*. Univariate analyses determined that every decrease of GFR by 10 mL/min/1.73 m^2^ significantly increased the risk of poor outcome (OR 1.19, 95% CI 1.09–1.30, *p* < 0.001) and mortality (OR 1.18, 95% CI 1.06–1.32, *p* = 0.002). In multivariate regression, adjusted for all variables with *p* value < 0.1, low GFR (GFR < 45 versus GFR equal to or more than 45) was associated with poor outcome (OR adjusted 2.15, 95% CI 1.01–4.56, *p* = 0.045).* Conclusion*. In IVT for acute stroke, renal dysfunction with GFR < 45 mL/min/1.73 m^2^ before treatment determined increased odds for poor outcome compared to GFR of more than 45 mL/min/1.73 m^2^.

## 1. Introduction

Intravenous thrombolysis (IVT) with recombinant tissue plasminogen activator (r-tPA) is an approved treatment for acute stroke reducing death and disability afterwards [1]. Chronic kidney disease (CKD) is associated with increased risk of stroke in the general population [2]. Its presence is an independent risk factor for a worse prognosis after a stroke [3, 4]. However in patients with renal impairment who were treated with IVT, prior reports are contradictory on the relationship between renal impairment and outcome. These studies have used various cutoff values for GFR for evaluating the association between renal insufficiency and outcome. Two studies used a cutoff of 90 mL/min/m^2^ [5, 6] while others set the cutoff at 60 mL/min/m^2^ [7–13]. Interestingly as recently reported in a large population-based study, in patients with renal insufficiency risk of cardiovascular morbidity and mortality rises sharply when GFR falls to less than 45 mL/min/m^2^ [14]. Therefore in this study we aimed to determine the effect of renal dysfunction with a new cutoff value (45 mL/min/m^2^) on the efficacy and safety of r-tPA for acute ischemic stroke patients in two experienced stroke centers in Turkey and Iran.

## 2. Method

### 2.1. Design

This study had a multicenter, hospital-based, and retrospective design. The effect of renal dysfunction was assessed with a cutoff value of 45 mL/min/1.73 m^2^ on outcome of stroke patients that received IVT.

Having an organized stroke database for acute stroke, Osmangazi University in Turkey (Medical Faculty, Eskisehir) and Imam-Reza Hospital in Iran (Tabriz University of Medical Sciences, Tabriz) were considered as study locations and the required data was selected from these databases. Consecutively, all patients who were treated with intravenous recombinant tissue plasminogen activator (r-tPA) from January 2009 to March 2015 were enrolled as participants in the study.

The following variables were collected for all patients: age, gender, initial stroke severity as assessed using the NIH Stroke Scale (NIHSS), systolic and diastolic blood pressure before IVT, stroke symptom onset to treatment time, initial creatinine and glucose levels in blood serum, previous treatment with antithrombotic agents, and stroke risk factors including hypertension, diabetes, atrial fibrillation, hyperlipidemia, history of stroke or TIA, and smoking. Functional outcome at 3 months was assessed by clinical visits or telephone calls using the modified Rankin Scale (mRS). Brain CT scans were done for all patients, immediately before and 24–36 hours after their treatment.

Patients were excluded if their serum creatinine values or 3-month mRS score follow-up data were not available. Patients who received interventional treatments (i.e., intra-arterial thrombolysis or mechanical thrombectomy) were excluded as well. All patients were treated according to AHA/ASA guidelines for management of patients with acute ischemic stroke, with 0.9 mg/kg of alteplase within 4.5 hours of symptoms onset (ASA 2010, 2013).

GFR was estimated by the Chronic Kidney Disease Epidemiology Collaboration (CKD-EPI) equation: GFR = 144 × (SCr/0.7)^−0.329^  × (0.993)^age^ (if female and SCr < or equal to 0.7 mg/dL), GFR = 144 × (SCr/0.7)^−1.209^  × (0.993)^age^ (if female and SCr > 0.7 mg/dL), GFR = 144 × (SCr/0.9)^−0.411^  × (0.993)^age^ (if male and SCr < or equal to 0.9 mg/dL), and GFR = 141 × (SCr/0.9)^−1.209^  × (0.993)^age^ (if male and SCr > 0.9 mg/dL) [15]. According to this equation, patients were divided into 2 groups, based on eGFRs: (1) a control group with GFR ≥ 45 mL/min/1.73 m^2^ and (2) a low GFR group with GFR < 45 mL/min/1.73 m^2^. None of the patients were receiving maintenance dialysis treatment. Post hoc, another category was added, very low GFR group defined as GFR < 30 mL/min/1.73 m^2^.

The major outcome measurements were as follows: poor functional 3-month outcome, defined as mRS scores 3 to 6, 3-month mortality rate, and symptomatic intracranial hemorrhage (SICH) according to criteria of the European Cooperative Acute Stroke Study II (ECASS-II) trial.

### 2.2. Statistical Analyses

Statistical analysis was performed using IBM SPSS statistics (version 21 for Windows); statistical test results were considered significant at *p* < 0.05. Data were presented as median and interquartile. Renal function evaluations, as measured by the eGFR, were compared with outcome measures as a categorical variable. The Fisher exact test or the* x*^2^ test was used for categorical variables. Due to nonnormal distribution of continuous variables, the Mann-Whitney *U* test was applied for analysis. The association between each GFR category and each outcome was estimated by calculations of odds ratios (ORs) with 95% confidence intervals (CIs) using binary logistic regression models. In multivariate analyses, models were adjusted for all variables with *p* value < 0.1 in univariate analyses, and variables were chosen by a backward Wald selection procedure using *p* < 0.10 in the likelihood ratio test for exclusion.

### 2.3. Ethical Consideration

This study was approved by the ethic committee of both localities.

## 3. Results

This study involved 426 patients with acute ischemic stroke that had been treated with IV r-tPA. Of these 426 patients, 14 did not have 3-month mRS follow-up and 9 did not have serum creatinine values so these patients were excluded from the study. Finally, 403 (94.6%) patients were determined as suitable for analysis. Among these eligible patients, 346 (85.9%) had a GFR of equal to or more than 45 mL/min/1.73 m^2^ (the control group) and 57 (14.1%) had a GFR of less than 45 mL/min/1.73 m^2^ (the low GFR group). Baseline characteristics of patients regarding GFR categories are shown in Table 1.

The median age of patients in the low GFR group was 8 years older (75 versus 67, *p* < 0.001); besides they more commonly had hypertension (*p* = 0.014) and had higher systolic blood pressures before treatment (*p* = 0.034). Baseline NIHSS scores were not significantly different between patients in the control group and those with low GFR [median (IQR); 15 (10–18) versus 16 (11–20), *p* = 0.243].

**Figure 1:**
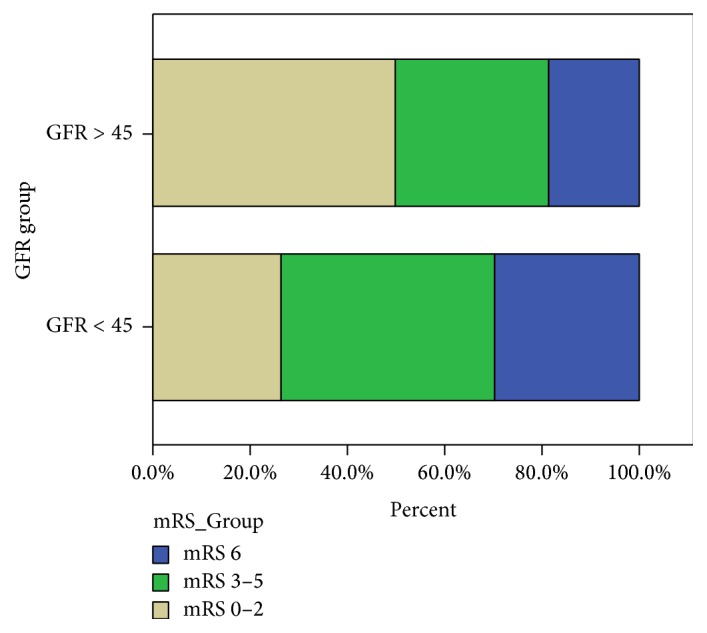
GFR related proportion of patients with unadjusted 3-month outcome.

At 3 months of follow-up, patients in the low GFR group had higher mRS scores than did those in the control group [median (IQR); 2.5 (1–5) versus 5 (2–5), *p* = 0.014]. Distribution of the unadjusted 3-month outcome stratified to the two GFR groups is presented in Figure 1.

As shown in Table 2, at 3 months, poor outcome and mortality were significantly more common in patients in the low GFR group than those in the control group (73.7% versus 50.0%; 29.8% versus 18.2%, resp.). Similarly, a favorable outcome (mRS 0-1) was significantly less common in the low GFR group (21.1% versus 43.6%). Comparison of SICH rates between the two groups (*p* value = 0.323) was determined as not statistically significant.

Univariate analyses of baseline characteristics of patients are shown in Table 3. In univariate analyses, every decrease of GFR by 10 mL/min/1.73 m^2^ showed a significant increase in the risk of poor outcome (OR 1.19, 95% CI 1.09–1.30, *p* < 0.001) and death (OR 1.18, 95% CI 1.06–1.32, *p* = 0.002).

In the multivariate analysis, models adjusted for all variables with *p* value < 0.1 are shown in Table 4. After adjustment, low GFR (GFR < 45 versus GFR of equal to or more than 45) was still associated with a poor outcome (OR adjusted 2.15, 95% CI 1.01–4.56, *p* = 0.045) but there was no longer an association between the low GFR group and mortality (OR adjusted 0.625, 95% CI 0.34–1.14, *p* = 0.625).

Post hoc analysis in patients with very low GFR (<30) versus the control group (GFR ≥ 45), after adjustment for all variables, is shown in Table 4 and demonstrates that the risk of poor outcome in patients with very low GFR (<30) was 12 times higher than that of patients in the control group (GFR ≥ 45) (OR adjusted 12.20, 95% CI 1.27–117.07, *p* = 0.030).

## 4. Discussion

In this retrospective study, eGFR of less than 45 mL/min/1.73 m^2^ was independently associated with poor outcome at the 3-month follow-up in patients who had received IV r-tPA. Previous reports on the relationship between renal dysfunction and the risk of a poor outcome were conflicting [5–13, 16–18]. This can be explained by the fact that these studies used different classifications of renal impairment, different definitions of poor outcome scores, different time periods of follow-up evaluations, different baseline characteristics of the studied objects, and different evaluation methods for renal function. Among these studies, nine used GFR of less than 60 mL/min/1.73 m^2^, two used GFR of less than 90 mL/min/1.73 m^2^, and one used creatinine of less than 1 mg/dL as the definition of renal dysfunction.

Risk of mortality and disability increases with a progressive decline in GFR. Recently a meta-analysis done by Hao evaluating 7796 stroke patients reported that renal dysfunction did not increase the risk of a poor outcome in patients who received thrombolysis [19]. Nevertheless, in Hao's study, the definition for renal insufficiency was considered as GFR < 60 in 7167 (91.9%) and GFR < 90 in 196 (2.5%) and proteinuria in 432 (5.5%) patients. However, Go and colleagues, in a large population-based study evaluating 1,120,295 adults over a median time of 2.8 years of follow-up, showed that the risk of cardiovascular events and mortality rose sharply for subjects with an eGFR < 45 mL/min/m^2^ [14]. The results of previous studies with a cutoff point of 60 or 90 mL/min/1.73 m^2^ may have had bias due to a dilution effect, because, in those studies, patients with a higher cardiovascular risk (i.e., GFR of less than 45) and patients with lower cardiovascular risk (i.e., GFR between 45 and 60) had been put in the same group and then compared with a normal GFR group (more than 60 or 90 mL/min/1.73 m^2^). Considering the latter study (Go), the cutoff point of 45 mL/min/1.73 m^2^ seemed to be more appropriate and, therefore, this value for GFR was applied instead of 60 or 90 mL/min/1.73 m^2^.

The mechanism which determines the link between outcome after a stroke and the effect of renal impairment remains unclear. In vitro studies have shown that patients with severe renal disease produce fibrin clots that are more compact, less permeable, and less susceptible to fibrinolysis [20, 21].

In this study, renal dysfunction was not associated with an increased risk of SICH. Similar results have been reported in most related studies [5–11, 16, 17, 22]. In the study reported by Gensicke et al., evaluation of 4,780 IVT-treated patients across 11 European stroke centers showed that SICH rate was higher in patients with low GFR according to univariate analyses; however it was not significant at multivariate logistic analysis [12].

Based on the results of this study and in conjunction with other studies, the higher rate of poor outcome in patients with renal dysfunction after IVT may not be related to occurrence of SICH. Renal dysfunction is associated with common vascular risk factors, including aging, hypertension, diabetes mellitus, dyslipidemia, and smoking. Interestingly, it has been shown that patients with renal dysfunction die from indirect causes rather than stroke. Besides, morbidity and mortality can frequently be attributed to other causes such as cardiac disease and susceptibility to infection [19].

A novel aspect of this study was application of a new cutoff point of 45 mL/min/1.73 m^2^ instead of 60 or 90 mL/min/1.73 m^2^ as it had been in previous studies. This study was retrospective in design so causality cannot be proven; however results demonstrate that renal insufficiency with GFR < 45 mL/min/1.73 m^2^ is an independent risk factor for long term poor outcome in patients who have received IV r-tPA and thereby might be considered as an important risk factor in future studies, in registrations, and even for developing associated guidelines.

Clinicians must act fast in the acute stroke setting to achieve effective and early recanalization. Hence, we evaluated renal functions based on GFR because it is a quick and practical test in patients who are candidate for IVT. Investigating albuminuria is an effective but time consuming test for acute stroke patients and may be considered in following hours or days after IVT. In further studies albuminuria with GFR should be analyzed.

This study has some limitations. Firstly, the present study did not have a control group and patients with renal failure who had not received IV r-tPA were not included in the study. Therefore, the effect of renal dysfunction on outcome between tPA-treated and non-tPA-treated patients could not be assessed. Secondly, this was an observational study and the associations reported in this article do not necessarily clarify causes. Thirdly, the sample size was relatively small. Fourthly, GFR calculations were made based on creatinine levels on admission, which may have been changed by the effects of an acute stroke. Therefore, a further study with a larger sample size, with a control group, and with consideration of a lower GFR cutoff is needed in future for more accurate assessment of the effect of renal insufficiency on stroke outcome.

In conclusion, in acute stroke patients receiving IVT, renal dysfunction with GFR < 45 mL/min/1.73 m^2^ before treatment is associated with increased odds of poor outcome in comparison to patients with GFR of more than 45 mL/min/1.73 m^2^.

## Figures and Tables

**Table 1:** Baseline characteristics of patients in the control and low GFR groups.

Baseline characteristics	Control group (GFR ≥ 45 mL/min/1.73 m^2^) (*n* = 346)	Low GFR group (GFR < 45 mL/min/1.73 m^2^) (*n* = 57)	*p* value
Age, years, median (IQR)	67 (56–73)	75 (67–77)	**p** ** value** < **0.001**
Male, *n* (%)	210 (60.7)	28 (49.1)	*p* value = 0.100
Hypertension, *n* (%)	217 (63.5)	42 (80.8)	**p** ** value** = **0.014**
SBP, mm Hg, median (IQR)	140 (120–160)	150 (130–170)	**p** ** value** = **0.034**
DBP, mm Hg, median (IQR)	80 (80–93)	90 (80–100)	*p* value = 0.150
Diabetes mellitus, *n* (%)	99 (28.9)	19 (36.5)	*p* value = 0.266
Atrial fibrillation, *n* (%)	99 (29.8)	16 (30.8)	*p* value = 0.889
Smoking, *n* (%)	117 (34.4)	7 (13.5)	**p** ** value** = **0.002**
Hyperlipidemia, *n* (%)	136 (40.2)	16 (30.8)	*p* value = 0.193
Previous use of antithrombotics, *n* (%)	84 (31.7)	13 (39.4)	*p* value = 0.374
Previous ischemic stroke, *n* (%)	31 (9.1)	8 (15.4)	*p* value = 0.160
Glucose, mg/dL, median (IQR)	129 (107–169)	139 (115–170)	*p* value = 0.390
Stroke severity, NIHSS, median (IQR)	15 (10–18)	16 (11–20)	*p* value = 0.243
Onset to treatment time, median (IQR)	150 (120–180)	147 (119–171)	*p* value = 0.466

DBP = diastolic blood pressure; GFR = glomerular filtration rate; IQR = interquartile range; NIHSS = National Institutes of Health Stroke Scale; SBP = systolic blood pressure.

**Table 2:** Unadjusted frequencies comparing outcomes between the two GFR groups.

	All *n* = 403	Control group (GFR ≥ 45) (*n* = 346)	Low GFR group (GFR < 45) (*n* = 57)	*p* value
Poor outcome, *n* (%)	215 (53%)	173 (50.0%)	42 (73.7%)	0.001
Mortality, *n* (%)	80 (19.9%)	63 (18.2%)	17 (29.8%)	0.042
Favorable outcome^*∗*^, *n* (%)	163 (40.4%)	151 (43.6%)	12 (21.1%)	0.001
SICH, *n* (%)	26 (6.5%)	24 (7%)	2 (3.5%)	0.323

^*∗*^Favorable outcome = mRS 0 or 1.

**Table 3:** Univariate analysis of clinical characteristics of all patients.

Baseline characteristics	Poor outcome (mRS 3–6)	Mortality	SICH
Age	1.04 (1.03–1.06) **p value < 0.001**	1.06 (1.03–1.09) **p value < 0.001**	1.02 (0.98–1.06) *p* value = 0.194
Sex	0.74 (0.50–1.11) *p* value = 0.157	0.89 (0.54–1.47) *p* value = 0.656	1.05 (0.47–2.36) *p* value = 0.889
Onset to time (each minute)	1.002 (0.998–1.006) *p* value = 0.332	1.001 (0.996–1.006) *p* value = 0.332	1.00 (0.99–1.01) *p* value = 0.163
NIHSS (each point)	1.24 (1.18–1.30) **p value < 0.001**	1.14 (1.08–1.21) **p value < 0.001**	1.06 (0.98–1.15) *p* value = 0.109
SBP (each mm Hg)	1.002 (0.998–1.006) *p* value = 0.640	1.005 (0.996–1.015) *p* value = 0.235	1.00 (0.98–1.01) *p* value = 0.806
DBP (each mm Hg)	1.002 (0.992–1.011) *p* value = 0.745	1.004 (0.992–1.016) *p* value = 0.495	1.01 (0.99–1.03) *p* value = 0.119
Glucose (each mg/dL)	1.007 (1.004–1.010) **p value < 0.001**	1.006 (1.003–1.009) **p value < 0.001**	1.004 (1.000–1.008) **p value = 0.028**
Diabetes mellitus	2.43 (1.54–3.82) **p value < 0.001**	1.37 (0.81–2.33) *p* value = 0.239	1.90 (0.83–4.32) *p* value = 0.125
Smoking	0.52 (0.34–0.81) **p value = 0.004**	0.72 (0.41–1.27) *p* value = 0.268	0.84 (0.34–2.07) *p* value = 0.708
Hyperlipidemia	0.87 (0.58–1.31) *p* value = 0.517	0.58 (0.33–1.02) **p value = 0.061**	1.20 (0.51–2.81) *p* value = 0.671
Hypertension	1.52 (1.00–2.31) **p value = 0.049**	1.25 (0.73–2.15) *p* value = 0.414	1.70 (0.66–4.37) *p* value = 0.414
Atrial fibrillation	1.45 (0.93–2.26) **p value = 0.095**	1.56 (0.92–2.66) *p* value = 0.101	1.18 (0.49–2.85) *p* value = 0.706
Previous stroke or TIA	0.83 (0.43–1.61) *p* value = 0.590	0.45 (0.15–1.31) *p* value = 0.147	0.37 (0.04–2.84) *p* value = 0.343
Previous use of antithrombotics	1.34 (0.82–2.19) *p* value = 0.690	1.44 (0.76–2.73) *p* value = 0.258	1.27 (0.51–3.19) *p* value = 0.598
Decreasing GFR (by 10 mL/min/1.73 m2)	1.19 (1.09–1.30) **p value < 0.001**	1.18 (1.06–1.32) **p value = 0.002**	1.05 (0.88–1.24) *p* value = 0.561

DBP = diastolic blood pressure; GFR = glomerular filtration rate; mRS = modified Rankin Scale; NIHSS = NIH Stroke Scale; SBP = systolic blood pressure; SICH = symptomatic intracranial hemorrhage.

Data are odds ratio (95% confidence interval).

**Table 4:** Multivariate analysis of major outcomes, low GFR (<45) versus control group (GFR ≥ 45). Odds adjusted for all variables with *p* < 0.1.

Baseline characteristics	Poor outcome (mRS 3–6)	Mortality	SICH
Age	1.03 (1.00–1.06) *p* value = 0.006	1.06 (1.02–1.09) *p* value = 0.001	NS
NIHSS (each point)	1.25 (1.18–1.32) *p* value < 0.001	1.12 (1.06–1.19) *p* value < 0.001	NS
Glucose (each mg/dL)	1.008 (1.004–1.011) *p* value < 0.001	1.007 (1.003–1.010) *p* value < 0.001	1.004 (1.000–1.008) *p* value = 0.028
Low GFR (<45)	2.15 (1.01–4.56) *p* value = 0.045	NS	NS
